# *Ex vivo* treatment of patient biopsies as a novel method to assess colorectal tumour response to the MEK1/2 inhibitor, Selumetinib

**DOI:** 10.1038/s41598-017-12222-9

**Published:** 2017-09-20

**Authors:** Sonia M. Novo, Stephen R. Wedge, Lesley A. Stark

**Affiliations:** 1 Edinburgh Cancer Research Centre and MRC Human Genetics Unit, Institute of Genetics and Molecular Medicine, University of Edinburgh, Western General Hospital, Crewe Rd, Edinburgh, Scotland EH4 2XU UK; 2 Northern Institute for Cancer Research, Newcastle University, Newcastle upon Tyne, NE2 4HH UK

## Abstract

Although an array of new therapeutics has emerged for the treatment of colorectal cancer, their use is significantly impacted by variability in patient response. Better pre-clinical models could substantially improve efficacy as it may allow stratification of patients into the correct treatment regime. Here we explore acute, *ex vivo* treatment of fresh, surgically resected human colorectal tumour biopsies as a novel pre-clinical model for identifying patient response to specific therapeutics. The MEK1/2 inhibitor, Selumetinib (AZD6244, ARRY-142886) was used as a tool compound. Firstly, we established an acute treatment protocol and demonstrated this protocol could differentiate phenotypic and pharmacodynamic responses to Selumetinib (0–3uM). We then used the protocol to evaluate Selumetinib response in tumours from 23 colon cancer patients. These studies revealed that the agent inhibited pERK1/2 phosphorylation in all tumours, caused a significant decrease in proliferation in 5/23 (22%) tumours, and that *KRAS/BRAF* mutant tumours were particularly sensitive to the anti-proliferative effects of the agent. These data are consistent with data from clinical trials of Selumetinib, suggesting that acute treatment of small tumour biopsies is worthy of further exploration as a pre-clinical model to evaluate colorectal cancer response to novel therapies.

## Introduction

Colon cancer remains a major cause of morbidity and mortality worldwide with one million diagnoses annually and at best, 60% survival^1^. Standard chemotherapy is associated with severe side effects and provides marginal benefit to the majority of patients^2^. Hence, there is an overwhelming need for novel, more targeted therapies that would impact on disease progression and patient outcomes.

A major impediment to progress in the development and use of targeted therapies for colorectal cancer is the lack of good pre-clinical models that can accurately predict the efficacy of new drugs and determine the patient population most likely to benefit^3^. Ideally, models should conserve the heterogeneity observed in patient tumours with respect to architecture, cell types (cancer and stromal) and tumour microenvironment. Traditional preclinical models of colorectal cancer, including tumour cell lines, genetically modified animals and cell line derived tumour xenografts, failed to capture this heterogeneity resulting in poor mapping to clinical outcomes^4,5^. Organoids derived from human tumours have recently gained traction as a representative model for colorectal cancer^6–8^. However, organoids do not recapitulate the heterogenous architecture of human cancers or model tumour-stromal interactions. Furthermore, the growth selection pressures applied during organoid generation has the potential to introduce bias. Another approach that is considered more representative is the use of patient derived xenografts (PDX), where a small fragment from a patient tumour biopsy is implanted either subcutaneously or orthotopically into an immunodeficient mouse^9,10^. However, the timescales and costs involved in this process are very significant. Development of alternative, innovative approaches for identifying tumours that will respond to specific agents is now essential if new therapies are to be targeted to the correct patient population.

*Ex-vivo* culture of intact tumour biopsies is potentially an extremely attractive system for analysing patient response to therapeutics as tumour architecture is retained, spatial relationships between tumour and stroma preserved, and the unique genetic and epigenetic modifications in each individual tumour incorporated^11–13^. Furthermore, biopsies from individual patients can be rapidly investigated. Supporting the validity of this model system, Vaira *et al*. demonstrated that vibrotome cut tumour sections grown in *ex vivo* culture are stable over time and exhibit the expected signalling response to PI3K inhibitors^14^. Furthermore, Rubio *et al*.^15^ demonstrated that the *ex vivo* response of fine needle pancreatic cancer biopsies to anti-cancer agents predicted the effects of the agents *in vivo* ^15^.

Here we used the MEK1/2 inhibitor, Selumetinib (AZD6244, ARRY-142886) as a model therapeutic to test *ex vivo* treatment of fresh, colorectal tumour biopsies as a method to determine patient sensitivity to new therapies. The RAS/RAF-MEK1/2-ERK1/2 signalling cascade is deregulated in approximately 50% of colorectal tumours, which drives tumour progression through constitutive ERK1/2 activity^16–18^. The most common cause for this deregulation is mutation in RAS/RAF family members, with mutant *KRAS* (40% of colorectal cancer cases) and *BRAF* (12% of cases) being the most prevalent^16,18,19^ Since MEK1/2 lies directly downstream of these mutations and uniquely phosphorylates ERK1/2, the MEK1/2 kinases have been identified as extremely attractive targets for therapeutic intervention.

Selumetinib is a potent, non-ATP competitive, small molecule inhibitor of MEK which has demonstrated significant anti-tumour activity against colorectal cancer *in vitro* in cell line studies and *in vivo*, in mouse xenograft models^20–22^. The agent has also demonstrated a manageable toxicity profile and evidence of disease stabilisation in Phase I and II clinical trials^23–27^. However, although promising, these studies have revealed considerable variability in response, with many tumours showing resistance^23–27^. Previous research aimed at stratifying patient sensitivity to the agent have primarily utilised human tumour cell lines, grown *in vitro* or as xenografts in nude mice. Not only have these approaches been poor at predicting outcomes in cancer patients but, they have also given inconsistent results with regards to the association between *ras*/*raf* mutations, pathway activation and drug sensitivity^20,28,29^. Clearly, alternative approaches are required for determining tumour sensitivity to this agent.

Here we developed an acute *ex-vivo* treatment protocol and demonstrated that target inhibition and phenotypic responses to Selumetinib could be quantified using this protocol. We also demonstrated a link between KRAS/BRAF mutational status and response to low dose Selumetinib, providing proof of principle that this approach can successfully identify genotype-phenotype association. We conclude that this methodology has the potential to identify patients that may benefit from treatment with MEK inhibitors and potentially other therapeutics and as such, warrants further clinical investigation.

## Materials and Methods

### Cell culture and reagents

Colon cancer cell lines RKO, HRT118, and HCT116 were obtained from ATCC/ECACC and maintained as described^30^. Selumetinib (AstraZeneca) was dissolved in DMSO (10 mM stock) and cells treated as specified.

### Acute *ex vivo* treatment of fresh colorectal tumour biopsies

Small colorectal tumour biopsies (1–2 cm^2^) were provided by a pathologist at the time of resection. Informed consent was obtained from all patients, full ethical approval was in place (Scottish Colorectal Cancer Genetic Susceptibility Study 3; Reference: 11/SS/0109- approved by the multicentre research ethics committee) and all procedures were performed in accordance with the relevant regulations. Biopsies were immediately placed in culturing media (MEM supplemented with glutamine, penicillin/streptomycin and anti-mycotic/antibiotic mix (1:100, Sigma) and transferred to the lab (within 1 hour). Following two PBS washes, tumours were placed in a petri dish of culturing medium and dissected into 1–2 mm^2^ fragments. Fragments were washed again with fresh media then placed in wells of a 96 well plate immersed in culturing medium containing 10% FCS and either 0, 0.1 or 3 µM Selumetinib. All samples had a final DMSO concentration of 0.03%. Two tumour fragments were treated per condition: one for immunohistochemical analysis and one for protein extraction. Samples were incubated at 37 °C for the times specified.

### Immunocytochemistry and immunohistochemistry

Immunocytochemistry and immunohistochemistry were carried out using standard protocols with antibodies against Ki-67 (1:100 DAKO) and active caspase-3 (1:100, BD Biosciences) and fluorescently tagged secondary antibodies (Jackson Labs)^31^.

### Imaging and analysis

Images were acquired as described^31^. The percentage of epithelial cells (defined by DAPI staining) positive for either Ki-67 or active caspase-3 was determined for five (immunohistochemistry) or ten (immunocytochemistry) random fields of view. In both cases, at least 190 cells per slide were analysed.

### Western Blot analysis

Whole cell lysates were prepared from cell pellets and frozen tumour samples (with the aid a QIAGEN TissueLyser LT) using 1x Lysis Buffer as per the manufacturer’s instructions (Cell Signalling). Western blot analysis was performed on 30 µg of protein using antibodies to phosphorylated p44/42 MAPK (p-ERK1/2) (1:1000; Cell Signalling), p44/42 MAPK (ERK1/2) (1:1000; Cell Signalling) and β-actin (1:2000, Sigma), as previously described^31^.

Immunoblots were quantified using ImageJ software. Relative pERK1/2 intensity was determined by subtracting background intensity from pERK1/2 band intensity then normalising this value using native ERK1/2 intensity and β-actin.

### Mutation analysis

Mutational analysis was performed by AstraZeneca using a Roche Cobas system. *KRAS* codon 12 and 13 mutations and *BRAF* ^V600E^ were investigated.

### Statistical analysis

P values throughout were calculated using a two-tailed Student’s *t*-test unpaired with equal variance. For immunohistochemistry, P values were derived by comparing the data from the five fields of view from the untreated sample to the five fields of view from the Selumetinib treated sample. Pearsons correlation coefficient (r^2^) was used to examine the relationship between fold decrease in proliferation in response to 0.1 µM Selumetinib compared to 3 µM Selumetinib, for the 5 patients that showed a significant response only at the 0.1 µM dose (Subset 2).

### Data availability

All data generated during this study, and all methods and materials utilised, are available upon request.

## Results

### Acute Selumetinib treatment inhibits MAPK/ERK signaling and induces a phenotypic response

We set out to determine whether an *ex vivo* treatment protocol could be used as a means of determining colorectal tumour sensitivity to Selumetinib. Firstly, we investigated the use of a number of methods that reportedly allow *ex vivo* growth of intestinal mucosa. However, we found that for all protocols tested, prolonged time in culture (>24 h) induced a significant increase in apoptosis and decrease in proliferation in colorectal tumour biopsies (Supplementary Table S1). Since ERK1/2 phosphorylation (which acts as a marker for MEK1/2 activity) is inhibited within hours of Selumetinib treatment in clinical studies, we postulated that sensitivity could be measured using *acute* (less than 6 h) drug exposure^23,24^.

**Figure 1:**
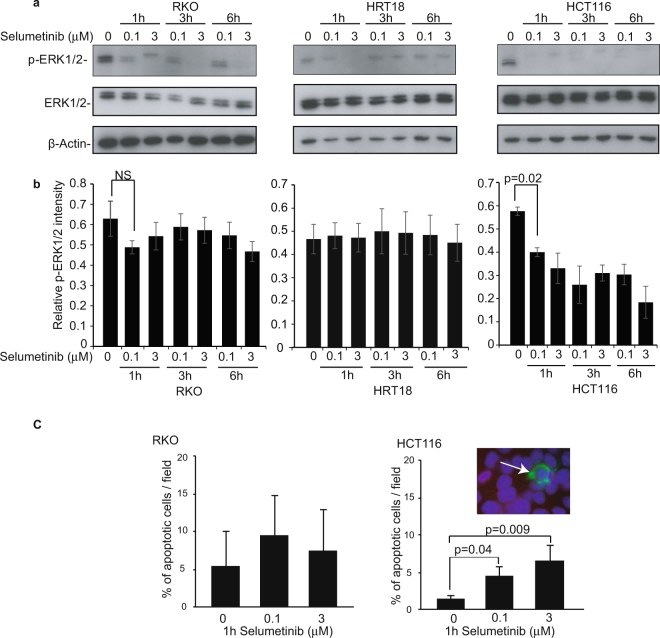
Acute Selumetinib treatment inhibits MAPK/ERK signaling and induces a phenotypic response in a panel of colorectal cancer cell lines. (**a**,**b**) Colon cancer cell lines RKO (BRAF mutant), HRT118 (KRAS mutant) and HCT116 (KRAS mutant) were treated acutely (0–6 h) with 0, 0.1 or 3 µM Selumetinib. (**a**) Top: Western blot analysis shows effects on ERK1/2 activity, as indicated by p-ERK1/2 levels. Native ERK1/2 and actin act as controls. Cropped gels are shown. Full length versions are in Fig. S4a. Bottom: Quantification of western blots was carried out using ImageJ as described in materials and methods. Graphs show mean (+/− s.e.m) relative p-ERK1/2 intensity at each dose. N = 3. (**b**) Immunocytochemistry with antibodies to active caspase-3 was used to monitor apoptosis. Insert: Immunomicrograph showing an apoptotic (active caspase-3 positive) cell. The percentage of positive cells was determined in at least ten fields of view (minimum 200 cells). Graphs show the mean +/− s.e.m. N = 3.

To test this principle, we firstly examined the pERK1/2 and phenotypic response to Selumetinib in colorectal cancer cell lines that have known sensitivity differences when exposed to the agent for prolonged periods; RKO (*BRAF* mutant) and HRT18 (*KRAS* mutant) that are resistant and HCT116 (*KRAS* mutant) that is sensitive^20,32^. The drug concentrations used in these studies were in keeping with those reported in patient plasma after Selumetinib treatment^23^. Figure 1a indicates that in all three cell lines tested, acute (0–6 h) exposure to pharmacologically relevant doses (0.1 or 3 µM) of Selumetinib induced a reduction in ERK1/2 phosphorylation. Notably, there was variability in this effect, with the reduction being most pronounced in the sensitive HCT116 cells (Fig. 1a). Immunocytochemistry with antibodies to Ki-67 and active caspase-3 indicated that effects on proliferation could not be detected within this time frame (data not shown). However, the pro-apoptotic effects of the agent were evident within 6 h of treatment in the sensitive HCT116 cell line (Fig. 1b).

**Figure 2:**
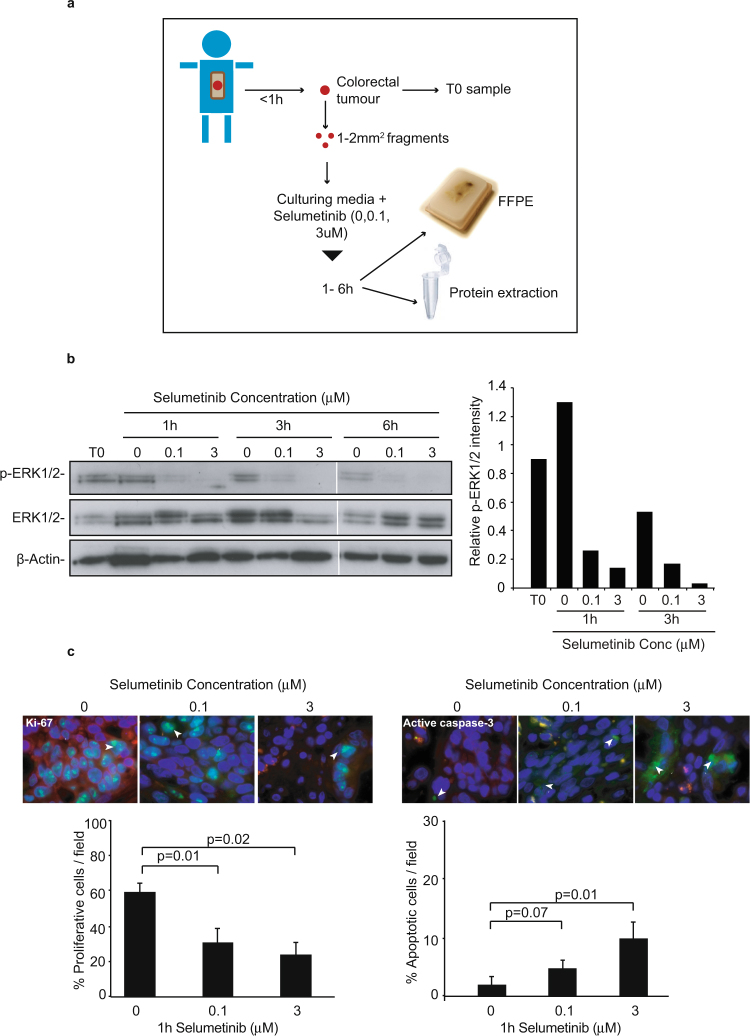
Short-term treatment of colorectal tumour biopsies ex vivo with Selumetinib. (**a**) Diagram depicting the short term *ex vivo* treatment protocol established. Figure adapted from O’Hara *et al*.^36^ with publishers permission. Three tumours were treated for 1, 3 and 6 h with 0–3uM Selumetinib. Data from one tumour (T10) is shown. (**b**) Western blot analysis, performed on whole tumour lysates with antibodies to p-ERK1/2, was used to analyse target inhibition in response to short term Selumetinib treatment. Native ERK1/2 and actin were used as controls. Cropped gels are shown. Full length versions are in Supplemental Fig. S4b. ImageJ was used to quantify relative P-ERK1/2 intensities as in Fig. 1. (**c**) Immunohistochemistry with antibodies to Ki-67 and active caspase-3 was used to monitor effects of acute Selumetinib treatment on proliferation and apoptosis respectively. The percentage of positive cells in five independent areas of tumour was quantified as described in materials and methods. Top: representative immunomicrographs. Bottom: Mean (+/− s.e.m) of the 5 fields (minimum 190 cells) for the 1 h time point. P values were calculated by comparing the five data points for the treated sample to the five data points for the non-treated sample using a two tailed Student’s t-test.

These data suggested that it may be worthwhile exposing human tumours to the agent for short periods and so, we established the *ex vivo* treatment protocol outlined in Fig. 2a to examine responses to 0, 0.1 or 3 µM Selumetinib in time course studies. We found that the agent inhibited ERK1/2 phosphorylation within 1 h of treatment and showed little further decrease after 3 or 6 h (Fig. 2b). Significant changes in proliferation and apoptosis were also quantifiable within a 1 h time frame (Fig. 2c). Therefore, given that tumour samples were limited in size, we proceeded by focussing on a 1 h treatment protocol.

### Acute treatment with Selumetinib inhibits MAPK/ERK signaling in human colorectal tumour explants

Tumours from 23 colorectal cancer patients were analysed using the optimised protocol. Non-cultured biopsies (time 0) acted as a baseline control. A summary of clinical, molecular and TNM staging data for the patient cohort is outlined in Table 1. The TNM stages equate to 2 stage IV, 8 stage III, 2 stage II and 4 stage I tumours. Given the small numbers, we did not distinguish by stage in further studies.

**Table 1:** Characteristics of patient cohort.

Sex (N = 23)	
Males	15 (65%)
Females	8 (35%)
**Age**	
Median	69
Range	39–80
**TNM staging** (**N** = **16**)	
T2	4 (25%)
T3	10 (62.5%)
T4	2 (12.5%)
N0	6 (37.5%)
N1	7 (43.75%)
N2	3 (18.75%)
M0	14 (87.5%)
M2	2 (12.5%)
**Site (N** = **16)**	
Colon	10 (62.5%)
Rectum	6 (37.5%)
**KRAS Status (N** = **22)**	
Wild Type	17 (77.3%)
Codon 12/13 mutant	5 (26.7%)
**BRAF Status (N** = **22)**	
Wild Type	20 (90.9%)
V600E Mutant	2 (9.1%)
**PI3K status**	
Wild Type	20 (90.9%)
Mutant	2 (9.1%)

**Figure 3:**
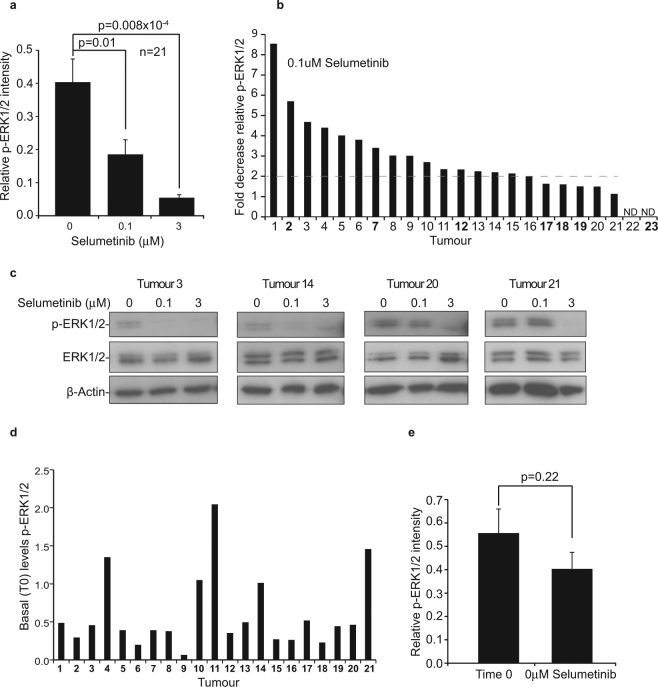
Effects of acute Selumetinib treatment on ERK1/2 activity in colorectal tumour biopsies. (**a**–**e**) Twenty three tumour biopsies were treated for 1 h *ex vivo* with 0, 0.1 or 3 µM Selumetinib, as outlined in Fig. 2a. Western blot analysis with antibodies to phosphorylated ERK1/2 (pERK1/2), native ERK1/2 and actin determined effects of the agent on relative ERK activity. A time 0 control was also analysed to gain information on baseline levels of active kinase. Quantification of western blots was carried out using ImageJ as described in materials and methods. (**a**) Graph showing mean (+/− s.e.m) relative p-ERK1/2 intensity at each dose N = 21. (**b**) Tumours were stratified based on fold decrease in relative pERK1/2 at 0.1 µM Selumetinib (compared to 0 µM). Stratified tumours were renumbered 1 to 23. ND = non-detectable at all doses. Those tumours mutant for KRAS or BRAF are highlighted in bold (see Fig. 5) (**c**) Example immunoblots. T3 and T14 have a > 2 fold decease in relative p-ERK1/2 levels following 1 h treatment with 0.1uM Selumetinib. T20 and T21 show minimal effect at this dose. Cropped gels are shown. Full length versions are in Supplemental Fig. S4c (**d**) Relative p-ERK1/2 levels at time 0 (basal) is shown for each tumour. (**e**) Effects of culture on relative p-ERK1/2 activity was determined by comparing intensity at T0 (basal) to 0uM (1 h culture) Mean of 23 samples (+/− s.e.m.) is shown.

Firstly, we used Western blot analysis with antibodies to phosphorylated and native ERK1/2, followed by ImageJ analysis of band intensity, to examine target inhibition. We found that collectively, short-term *ex vivo* exposure to Selumetinib caused a significant, dose-dependent decrease in relative ERK1/2 activity in all colorectal tumour biopsies (Fig. 3a). Individually, all tumours demonstrated significant target inhibition (>2 fold decrease in relative pERK1/2) when exposed to 3 µM Selumetinib. Target inhibition (>2 fold) was also observed in the majority (16/21) of tumours upon exposure to 0.1 µM Selumetinib (Fig. 3b and c). However, there were tumour specific differences in the degree of this effect (Fig. 3b and c).

In keeping with previous studies, we found that the variability in target inhibition at 0.1 µM Selumetinib was not related to basal ERK1/2 activity (Fig. 3d). It was also unrelated to the stress of being placed in culture. Indeed, we found that culturing had a minimal effect on the relative activity of the kinase (determined by comparing pERK1/2 levels at Time 0 and 0 µM) (Fig. 3e). Together, these data indicate that acute *ex vivo* exposure to Selumetinib induces a robust target inhibition in colorectal tumours and suggest that this may be a viable approach to examine tumour specific responses to treatment.

### Acute *ex vivo* treatment with Selumetinib identifies tumour subsets with distinct phenotypic responses

**Figure 4:**
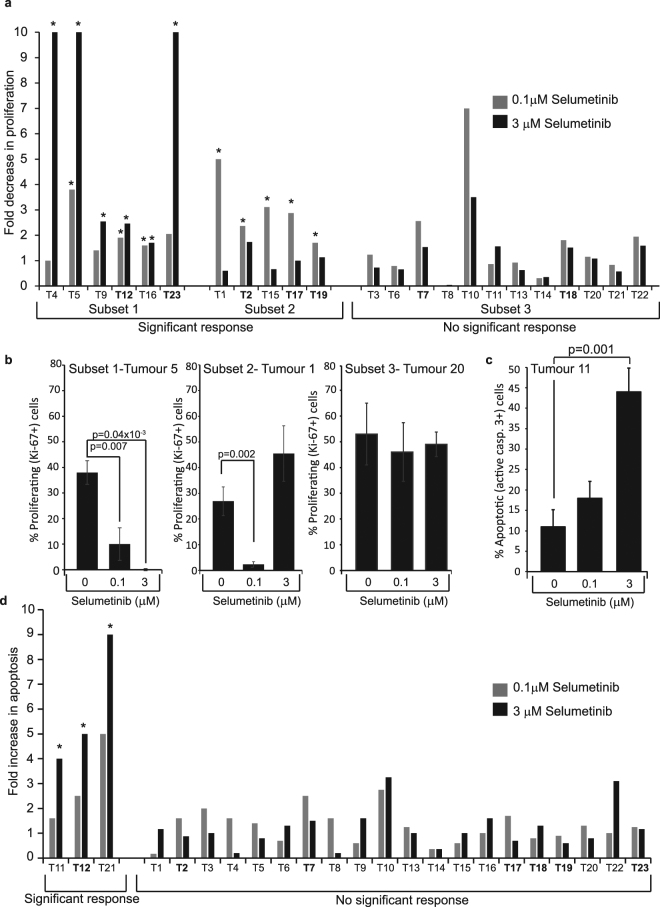
Proliferation and apoptosis in colorectal tumour biopsies following acute Selumetinib *treatment*. (**a**–**d**) Tumour biopsies from 23 colorectal cancer patients were treated with 0, 0.1 or 3 µM Selumetinib for 1 h as detailed in Fig. 2a. Immunohistochemistry was performed on formalin fixed, paraffin embedded tissue with antibodies to active caspase-3 (marker for apoptotic cells) and Ki-67 (marker for proliferating cells). The percentage of positive cells was quantified in five independent areas of tumour for each sample and each treatment point (as in Fig. 2). Significance was determined by comparing the five data points for the treated sample to the five data points for the non-treated sample using a two tailed Student’s t-test. (**a** and **d**) Graphs showing fold change in proliferation (**a**) and apoptosis (**d**) for all tumours at both doses (compared to 0 µM). *P < 0.05 when significance was determined from raw data as described above. Those tumours mutant for KRAS or BRAF are highlighted in bold (see Fig. 5). (**b**,**c**) Graphs showing the mean percentage of proliferating (**b**) or apoptosing (**c**) cells in the 5 independent fields (+/− s.e.m) for representative sensitive and resistant tumours.

Next, immunohistochemistry was used to determine whether differential proliferative (Ki-67) and apoptotic (caspase-3) responses to Selumetinib could be detected using acute drug treatment of small tumour biopsies. For each tissue section, the percentage of positive cells in five fields of view (>190 cells total) was quantified. The average of these five fields was then calculated and used to determine the fold decrease in proliferation/increase in apoptosis (compared to 0 µM) for each tumour at both doses (Fig. 4a and d). To determine whether a response was statistically significant, the % proliferation/apoptosis for the five fields of view for treated samples was compared to the five fields of view for 0 µM (using a students Ttest). Tumours showing a significant (p < 0.05) response at either dose were deemed sensitive. Examples of the raw data for sensitive and resistant tumours are shown (Fig. 4b and c).

These studies revealed that, taking both doses into account, *ex vivo* exposure to Selumetinib significantly (p < 0.05) inhibited proliferation in 11/23 (47.8%) tumours (Fig. 4a and b). Interestingly, within this group of sensitive tumours there were two distinct subsets. One subset (6/22) demonstrated a significant decrease at 3 µM but not necessarily 0.1 µM Selumetinib (Fig. 4a and b). In the other sensitive subset (5/22), a significant effect was *only* apparent at the lower dose of the agent (Fig. 4a and b). Indeed, in this subset there was an inverse correlation (r^2^ = −0.66) between response at 0.1 µM and that at 3 µM. On analysis of apoptotic effects we found that in 3/23 tumours there was a dose-dependent increase in apoptosis within an hour of Selumetinib treatment. However, in contrast to the anti-proliferative effect, this only reached significance at the higher dose (3 µM) of the agent (Fig. 4c and d).

We did consider that the acute phenotypic response to Selumetinib could be influenced by initial rates of proliferation or apoptosis. However, we found that on a tumour by tumour basis, there was no association between basal proliferative or apoptotic state (determined by the Time 0 sample) and Selumetinib response (Supplemental Figs S1 and 2). We also considered that the acute phenotypic response may be influenced by the degree of catastrophe when the biopsy was placed in culture. However, again we found no association between maintenance of tissue integrity in culture (determined by comparing Time 0 to 0 µM) and the anti-proliferative/pro-apoptotic effects of Selumetinib (Supplemental Figs S1 and 2).

Taken together, these data suggested that acute *ex vivo* treatment of small tumour biopsies can identify the intrinsic phenotypic response to Selumetinib in colorectal tumours.

### Acute treatment confirms that KRAS mutations confer increased sensitivity to the anti-proliferative effects of Selumetinib

Given the differential patient responses to Selumetinib in phase I clinical trials, efforts have been focussed on identifying predictive molecular biomarkers of sensitivity or resistance. Since our *ex vivo* culture system revealed sensitive and resistant tumours, we next determined whether it could be used to identify biomarkers of response. Firstly, we assessed the association between phenotypic response and basal levels of p-ERK1/2, or inhibition of ERK1/2 activity. We would predict that tumours with high basal pERK1/2 would be more dependent on the RAF/MEK/ERK pathway for growth and survival, and hence, more sensitive to the MEK inhibitor. However, we found that tumours that showed high basal levels of pERK, such as tumour 11, showed no phenotypic response to the agent (Figs 3d and 4c). Conversely, tumours such as T5, which showed a significant, dose-dependent, anti-proliferative response to Selumetinib, had low basal pERK1/2 (Figs 3d and 4b). Indeed, when basal pERK1/2 was compared to fold response at 0.1 or 3 µM Selumetinib, there was no correlation. Similarly, there was no correlation between degree of target inhibition and phenotypic response (see tumour 14 for example (Figs 3 and 4)).

Tumours with mutations in the MEK/ERK pathway have been found to be particularly sensitive to Selumetinib therapy. However, some disparate results have been reported^20,32–34^. Therefore, we next determined whether our model could be used to explore genotype, phenotype relationships. Firstly, our panel of tumours were evaluated for the most common *KRAS* (*codon 12 and 13*) and *BRAF* (V600E) mutations. Mutations in *PIK3CA*, which are reported to increase resistance to MEK inhibition, were also evaluated^19^ (Table 1). From the 22 samples analysed, five *KRAS (codon 12 and 13)*, two *BRAF(V600E)* and 2 *PIK3CA* mutants were identified, including one tumour with a *BRAF* and *PIK3CA* mutation (supporting Fig. 3).

**Figure 5:**
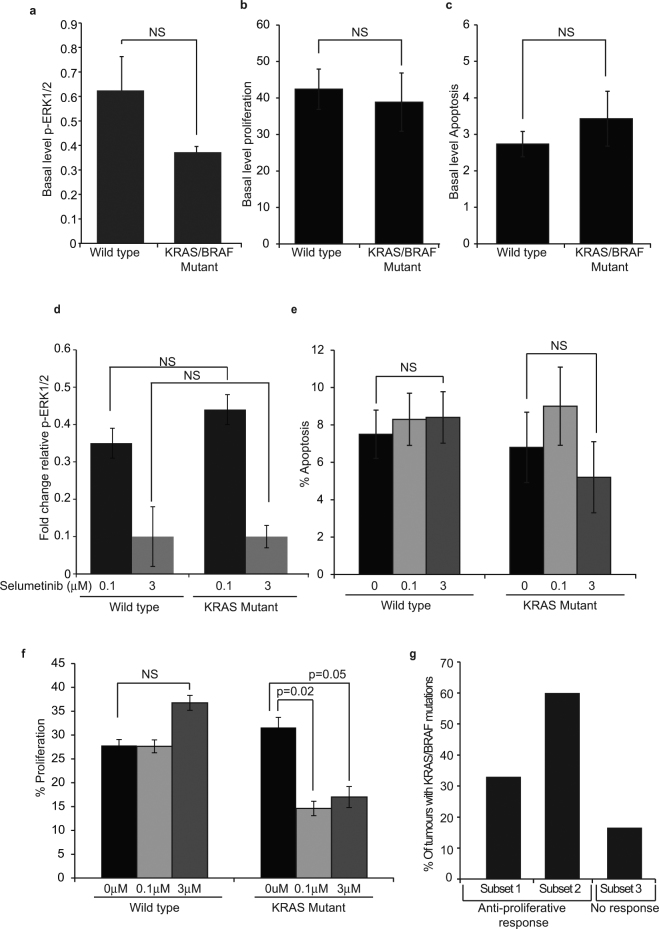
Influence of KRAS mutational status on ERK1/2 activity and tumour sensitivity to Selumetinib. (**a**–**f**) Twenty two tumours were analysed for codon 12/13 mutations in KRAS, and for BRAFV600E mutations. (**a**–**c**) Graphs showing mean (+/− s.e.m) basal (T0) levels of (**a**) p-ERK1/2, (**b**) proliferation and (**c**) apoptosis in wild type and KRAS/BRAF mutant tumours collectively. See also Supporting Fig. 3. (**d**) Fold change in relative p-ERK1/2 activity in wild type and KRAS mutant tumours. (**e and f**) Mean (+/− s.e.m, N = 22) percentage (**e**) apoptosis and (**f**) proliferation in untreated (0 µm) and treated (0.1–3 µM Selumetinib) wild type and KRAS mutant tumours. (**g**) Subsets 1, 2 and 3 refer to subsets 1, 2 and 3 outlined in Fig. 4. Subset 1-A significant decrease in proliferation in response to 3 µM Selumetinib but not necessarily 0.1 µM. Subset 2- Significant response only at 0.1 µM Selumetinib. Subset 3: No significant response.

First, we compared basal levels of p-ERK1/2 and found that there was no significant difference between wild type and *KRAS/BRAF* mutant tumours, either when mutants were analysed collectively (all mutations in the pathway) or when mutations were considered individually (i.e *KRAS* codon 12/13 and *BRAF* ^V600E^) (Fig. 5a and Supplemental Fig. S3a). There was also no significant difference in the basal rates of proliferation or apoptosis in the two populations (Fig. 5b,c and Supplemental Fig. S3b and c). Upon Selumetinib treatment, we found that target inhibition and the induction of apoptosis did not differ between wild type and *KRAS* mutant tumours (Fig. 5d and e). However, the agent had dramatically different effects on proliferation in the two populations. We found that Selumetinib induced a significant reduction in proliferation specifically in *KRAS* (codon 12 and 13) mutant tumours (Fig. 5f). Furthermore, this reduction was particularly pronounced at the lower dose (0.1 µM (p = 0.02) vs 3 µM (P = 0.05)) of the compound (Fig. 5f). In fact, 3 of the 5 tumours (60%) that showed a significant anti-proliferative response uniquely at 0.1 µM Selumetinib (Subset 2) had *KRAS* or *BRAF* mutations (Fig. 5g). In contrast, mutations were only detected in 2 of the 12 (16.7%) resistant tumours (Fig. 5g).

Collectively, these data suggest that tumours with *KRAS* mutations have increased sensitivity to the anti-proliferative effects of Selumetinib. They also provide proof of principle that this model system can detect specific anti-proliferative effects and may be used to establish associations between genetic biomarkers and phenotypic responses to drug treatment.

## Discussion

Targeted therapy, based on the unique biological characteristics of each individual tumour, is revolutionizing treatment options for cancer patients. However, to realise the full clinical potential of this approach, better tools are required for identifying biologically discreet subpopulations of patients, so that they can be stratified to correct therapeutic regimes. Here we explored the use of acute *ex-vivo* treatment of small, tumour biopsies as a novel method for identifying colorectal tumour sensitivity to specific therapeutics. The MEK inhibitor, Selumetinib, was used to test the efficacy of the approach.

Using the short term *ex-vivo* treatment protocol we established that: 1) 3 µM of Selumetinib inhibits pERK1/2 phosphorylation in all tumours and the effect is independent of whether or not tumours harbour a *KRAS* or *BRAF* mutation. 2) Low dose Selumetinib induced a significant reduction in Ki-67 staining in 5/23 (22%) tumours. 3) the phenotypic responses to the agent were independent of the magnitude of target inhibition, and 4) *KRAS* mutant tumours were particularly sensitive to the anti-proliferative effects of Selumetinib. These findings are consistent with those of a phase I clinical study where it was shown that 5/20 biopsies of human tumours demonstrated a 2 fold reduction in Ki-67 staining after 7 days Selumetinib treatment, that all biopsies demonstrated a reduction in pERK1/2 staining, that wild-type and *KRAS*/*BRAF* mutants showed similar levels of target inhibition and that mutant tumours were more sensitive to the growth inhibitory effects of the agent^23^. Using xenografts of patient derived metastatic colorectal cancer grown in mice, Migliardi *et al*., also found that Selumetinib inhibited pERK1/2 activity in all cancers and caused disease stabilisation in 27%^29^. These similarities suggest that our *ex vivo* model system may have the potential to reveal clinical responses to MEK inhibitors. It would now be extremely interesting to undertake clinical trials alongside *ex vivo* treatment in order to fully assess this approach.

Our data indicate that mutations in the MAPK/ERK pathway are associated with an enhanced anti-proliferative response to low dose Selumetinib. This result was extremely encouraging with regards to the model system as it suggested the phenotypic effects we observed at low doses of the agent are valid, and that this approach has the potential to identify genotype-phenotype relationships. However, these data are in contrast to a number of studies in the field, which demonstrate the phenotypic response to Selumetinib is independent of the mutational status of this pathway^32,34^. One possibility for the discrepancy is the dose of the agent utilised. We found that the relationship between genotype and phenotype was particularly evident at 0.1 µM Selumetinib, which is a tenth of the maximum tolerated dose in humans and is a lower dose than is generally used in *in vitro* studies. Since resistance to MEK inhibitors in RAS/RAF mutated tumours has been attributed to negative feedback mechanisms, it is possible that at this lower dose, MEK inhibitors show more specificity for their target and are less likely to activate these feedback mechanisms. Our *ex-vivo* methodology would be an ideal platform to interrogate such adaptive signalling further.

In this study we used a MEK inhibitor as a model therapeutic to test our *ex vivo* methodology. However, the protocol we established has the potential to reveal information regarding response to other agents. Indeed, we have used this protocol to examine tumour response to low doses of the chemopreventative/therapeutic agent, aspirin. Our cell line studies had demonstrated that aspirin causes an increase in levels of the NF-κB suppressor, COMMD1^35^. Using the described *ex vivo* protocol with patient tumour biopsies, we could show that aspirin also caused increased COMMD1 in 3/4 colorectal tumours, again potentially identifying patients that would benefit for aspirin therapy^36^. Together, these data suggest that this approach has broad application.

Although other groups have published protocols for assessing tumour response to therapeutics *ex vivo*, these generally involve tumour manipulation (for example by sectioning or xenograft growth) prior to *ex vivo* culture^14,15^. The advantage of our method is that it does not require pre-manipulation, enabling the use of very fresh, extremely small tumour biopsies. As we have shown drug response (Selumetinib and aspirin) can be quantified after acute treatment using this *ex vivo* culture protocol, this methodology could be ideal as a fast, cost effective means of identifying patients that would benefit from Selumetinib or other therapies following surgical resection of primary tumour. Since this protocol does not allow long term growth of tumours, it could be argued that patients who respond with delayed kinetics may be overlooked. The question of whether the kinetics of the response influences the phenotypic outcome is interesting and should be addressed in clinical trials undertaken alongside *ex vivo* treatment.

In summary, we show that acute *ex vivo* treatment of small biopsies of human colorectal tumours has considerable potential as means to reveal patient specific responses to MEK inhibitors and other therapeutic agents and to identify markers of response. We conclude that this approach warrants further investigation.

## Electronic supplementary material

Supporting data
