# Supplementary material for: Ex vivo treatment of patient biopsies as a novel method to assess colorectal tumour response to the MEK1/2 inhibitor, Selumetinib
Source: Sci Rep. 2017 Sep 20;7:12020. doi: 10.1038/s41598-017-12222-9 (PMC5607258; doi:10.1038/s41598-017-12222-9)

Ex vivo treatment of patient biopsies as a novel method to assess colorectal tumour response to the MEK1/2 inhibitor, Selumetinib

Sonia M. Novo; Stephen R. Wedge and Lesley A. Stark

Supplementary table 1. Growth of colorectal tumours ex vivo. Table documenting the methods explored to grow colorectal tumour biopsies in culture. References for specific protocols are provided along with the number of tumours that were cultured using each protocol, the number of days the tumours were monitored for and the outcome.

Supporting Figure 1 . Anti-proliferative response to Selumetinib is independent of basal rates of proliferation or culturing effects. Tumour biopsies from 23 colorectal cancer patients were fixed immediately (Time 0) or treated with 0, 0.1 or 3uM Selumetinib for 1h as detailed in Fig 2a. Immunohistochemistry was performed on formalin fixed, paraffin embedded tissue with antibodies to Ki-67. The percentage of positive cells was quantified for five independent fields of view (as outlined in materials and methods) for each sample. Tumours were divided into subgroups based on their response to Selumetinib (See Fig. 4). (a) The mean (from 5 fields of view) basal (T0) levels of proliferation are shown. (b) Culturing effects were determined by comparing proliferation at T0 and 0uM (1h in culture).

Supporting Figure 2 . Apoptotic response to Selumetinib is independent of basal rates of apoptosis or culturing effects. Tumour biopsies from 23 colorectal cancer patients were fixed immediately (Time 0) or treated with 0, 0.1 or 3uM Selumetinib for 1h as detailed in Fig 2a. Immunohistochemistry was performed on formalin fixed, paraffin embedded tissue with antibodies to active caspase-3 (apoptotic marker). (a) and (b), the basal rates of apoptosis and culturing effects on apoptosis were determined as for proliferation in Fig S1.

Supporting Figure 3 . Basal rates of p-ERK activity, proliferation and apoptosis are independent of KRAS/BRAF mutational status. Twenty two tumours were analysed for codon 12/13 mutations in KRAS and for BRAFV600E (a) to (c) Basal (T0) rates of relative p-ERK, proliferation and apoptosis were determined as for Figs 3 and 4.

Supporting Figure 4. Full length western blots for figures 1 to 3. Immunoblots were performed with the indicated antibodies on the indicated cell lines/tumours. Hatched boxes

highlight cropped areas. (a) Colon cancer cell lines RKO (BRAF mutant), HRT118 (KRAS mutant) and HCT116 (KRAS mutant) were treated acutely (0-6h) with 0, 0.1 or 3uM Selumetinib. Western blot analysis shows effects on ERK1/2 activity, as indicated by p-ERK1/2 levels. Native ERK1/2 and actin act as controls. See also Fig.1. (b) Tumour T10 was treated *ex vivo* with 0-3uM Selumetinib for 1, 3 and 6h. Western blot analysis was performed on whole tumour lysates with the indicated antibodies. See also Fig. 2. (c) Example immunoblots from patient tumours treated with 0-3uM Selumetinib for 1h. T3 and T14 have a >2 fold decease in relative p-ERK1/2 levels following 1h treatment with 0.1uM Selumetinib while T20 and T21 show minimal effect at this dose. See also Fig. 3.

Supplementary table 1

Method	Reference	Number of tumours	Time in culture	Outcome
Stainless steel mesh / Floating	Based on Browining et al (1969) and Autrup et al (1980)	5	4 Days	Tissue damage due to daily changes into new plates with fresh media.
Filter Paper	Hearn et al (1999)	13	30 Days	High apoptosis rates observed at 24h and thereafter. Lack of tissue regeneration.
Type I collagen gels	Freeman et al (1986) Vescio et al (1987)	23	47 Days	Consistent invasion of epithelial cells in collagen gels. High apoptotic rates observed in tumour bodies. Lack of response to treatment with growth inhibitor agents.
Multicellular tumour spheroids	Kondo et al (2011)	6	several weeks	Spheroid formation consistently observed after 24h in culture. Method is technically challenging and time consuming so not applicable to therapeutic assay

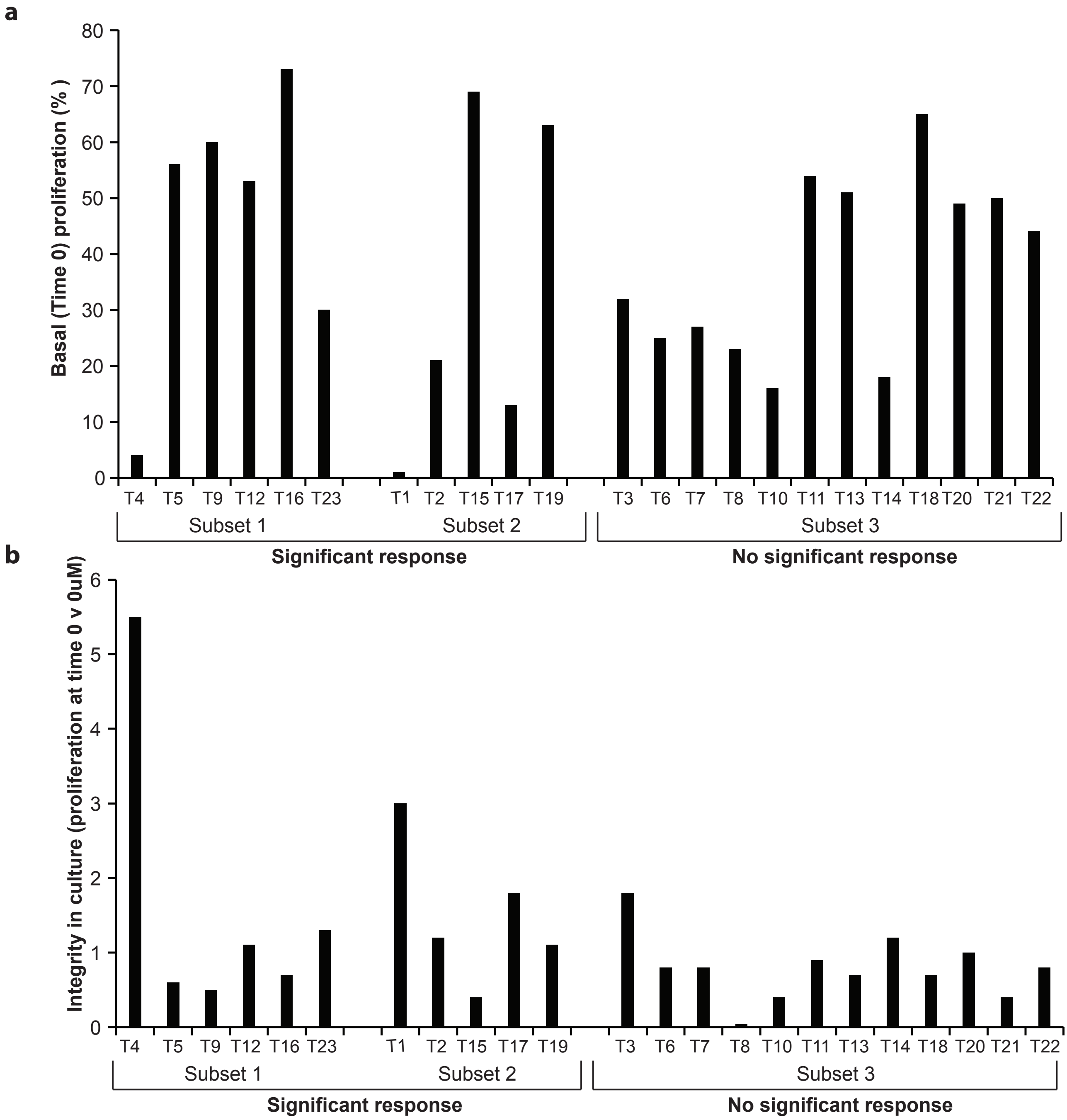
a
b
Basal (Time 0) proliferation (%)
Integrity in culture (proliferation at time 0 v 0uM)
T4
T5
T9
T12
T16
T23
T1
T2
T15
T17
T19
T3
T6
T7
T8
T10
T11
T13
T14
T18
T20
T21
T22
Subset 1
Subset 2
Subset 3
Significant response
No significant response

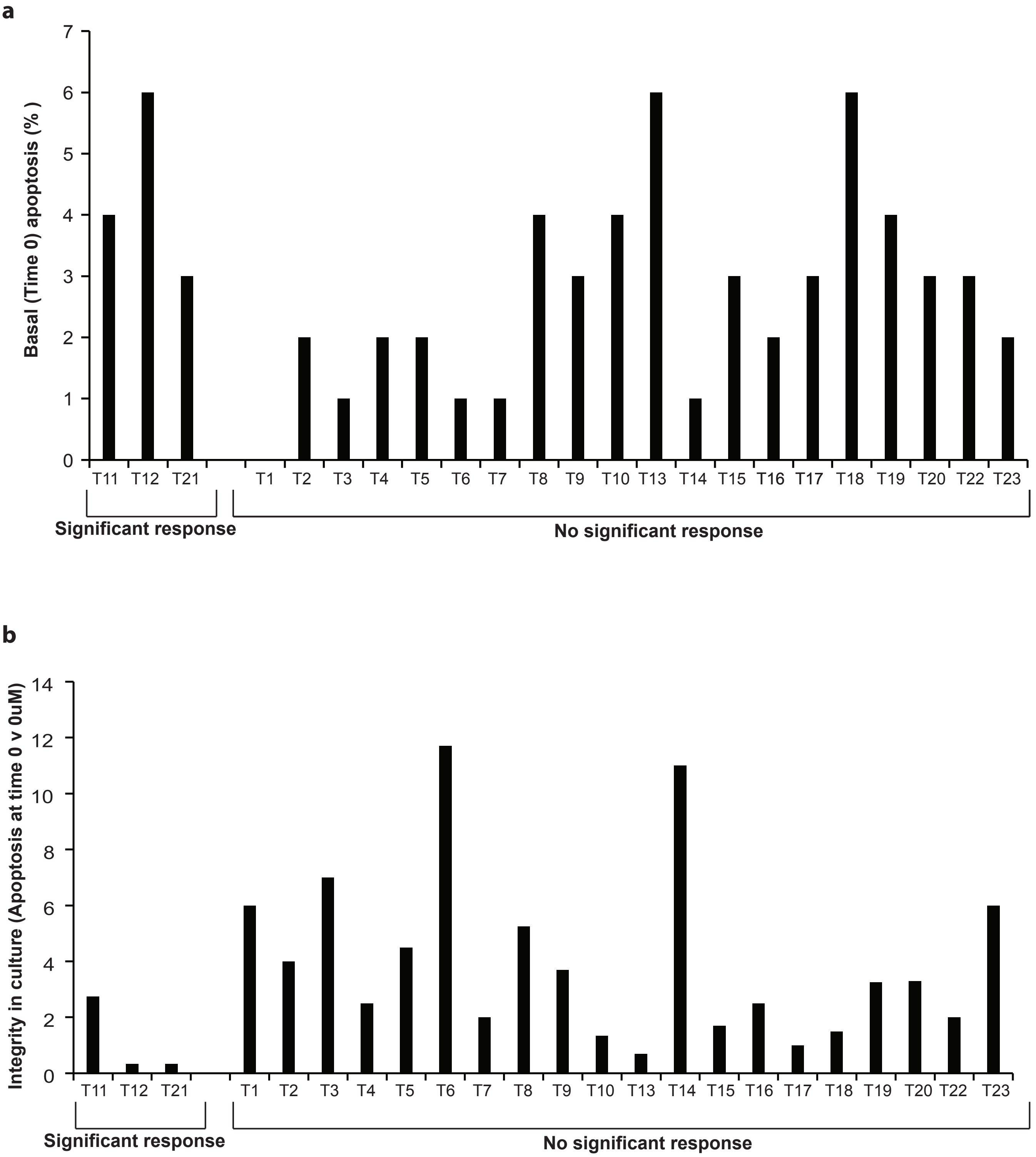
a
Basal (Time 0) apoptosis (%)
0
1
2
3
4
5
6
7
T11 T12 T21 T1 T2 T3 T4 T5 T6 T7 T8 T9 T10 T13 T14 T15 T16 T17 T18 T19 T20 T22 T23
Significant response
No significant response
b
Integrity in culture (Apoptosis at time 0 v 0uM)
0
2
4
6
8
10
12
14
T11 T12 T21 T1 T2 T3 T4 T5 T6 T7 T8 T9 T10 T13 T14 T15 T16 T17 T18 T19 T20 T22 T23
Significant response
No significant response

Supporting figure 3

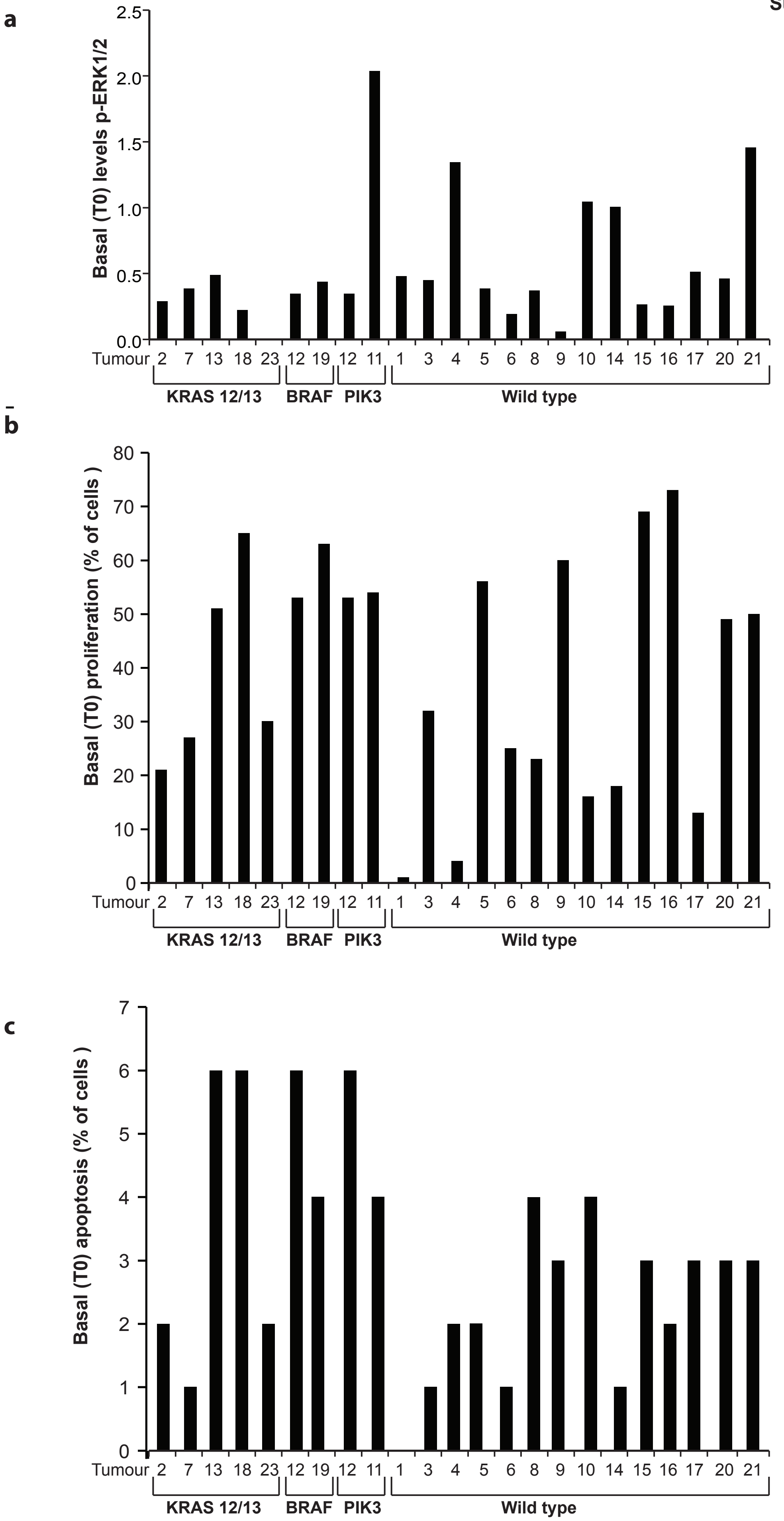

Supporting figure 4

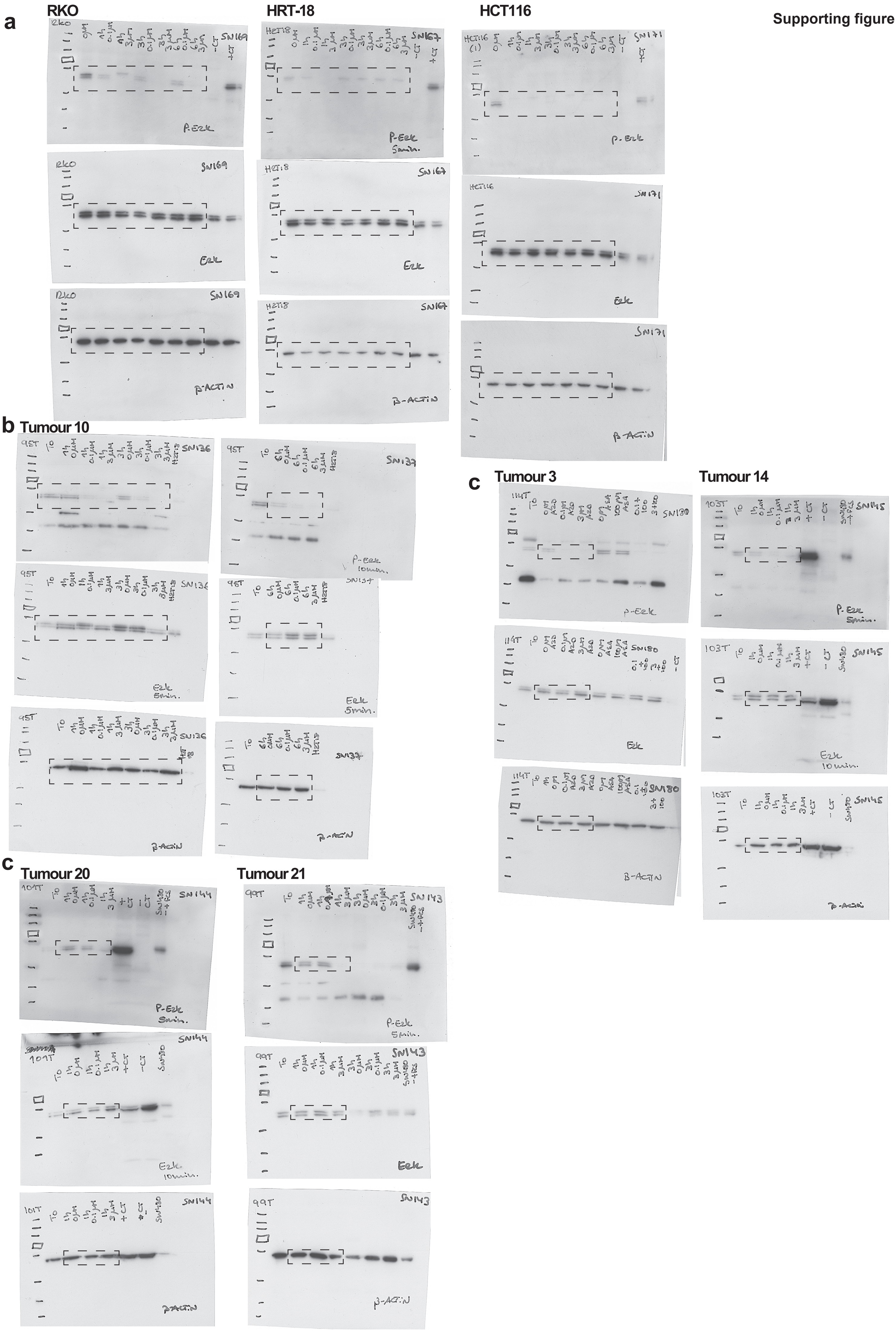

Supplement: Supplementary file 1 — Supporting data [file 41598_2017_12222_MOESM1_ESM.pdf]
